# Effects of exercise interventions on hand-eye coordination and fine motor skills in children with developmental coordination disorder: a meta-analysis

**DOI:** 10.3389/fphys.2025.1689256

**Published:** 2025-10-17

**Authors:** Shi Wenying, Hou Yaoqi, Zhou Shenning, Song Xiangqin

**Affiliations:** Beijing Normal University College of Physical Education and Sports, Beijing, China

**Keywords:** developmental coordination disorder, hand-eye coordination, fine motor skills, exercise, meta-analysis

## Abstract

**Objective:**

This meta-analysis was conducted to examine the efficacy of exercise interventions in improving hand-eye coordination and fine motor skills in children with developmental coordination disorder (DCD), thereby providing evidence-based support for clinical management strategies targeting these domains.

**Methods:**

Four electronic databases (PubMed, Web of Science, Cochrane Library, Embase) were systematically searched for relevant literature published from inception until 18 July 2025. Two researchers independently assessed the quality of included studies using the Cochrane risk-of-bias tool. The inclusion criteria include: children (<12 years old) who have been diagnosed with DCD; The intervention measures were any form of physical activity, while the control group received routine care or waiting; Primary outcome measures included the Movement Assessment Battery for Children (M-ABC), the Bruininks-Oseretsky Test of Motor Proficiency, Second Edition (BOT-2), the Beery-Buktenica Developmental Test of Visual-Motor Integration (Beery VMI), and the Test of Motor Impairment (TOMI). Data synthesis and statistical analyses were performed using RevMan 5.4 and Stata 18.0 software.

**Results:**

A total of 14 literature were included in the meta-analysis, all of which were in foreign languages, with a total sample size of 528 cases. The results of the meta-analysis showed that motor intervention could improve hand-eye coordination (SMD = 0.45, 95%CI: 0.16-0.73, P = 0.002) and fine motor skills(SMD = 0.74, 95%CI: 0.3-1.18, P = 0.001) in children with DCD, and the difference in treatment outcomes between the experimental group and the control group was statistically significant (P < 0.05). The results of subgroup analysis showed that moderate to high intensity hand-eye coordination and fine motor skills improved by moderate and large effects respectively (P < 0.01), and both hand-eye coordination and fine motor skills improved by large effects when the total intervention duration was over 720 min (P < 0.05).

**Conclusion:**

Exercise intervention can effectively improve hand-eye coordination and fine motor skills in children with DCD. However, the magnitude of its therapeutic effect may be affected by the intensity of exercise and the total duration of intervention. Through a systematic exercise program, hand-eye coordination and fine motor skills in children with DCD can be better improved.

**Systematic Review registration:**

identifier CRD420251118109.

## 1 Introduction

Developmental coordination disorder (DCD) is a prevalent neurodevelopmental condition in children (De Roubaix et al., 2024), characterized by motor coordination performance substantially below expected levels for chronological age (Tran et al., 2022; Ke et al., 2023). These impairments are not attributable to intellectual disabilities, neurological disorders, or acquired brain injuries. Children with DCD frequently exhibit marked clumsiness in daily activities, academic tasks, and social interactions, manifesting as difficulties in shoe-tying, handwriting, utensil use, or sports participation (Biotteau et al., 2019). According to the *Diagnostic and Statistical Manual of Mental Disorders (fifth ed.; DSM-5)*, diagnostic criteria for DCD include delayed motor skill acquisition that significantly interferes with academic achievement, occupational performance, or activities of daily living (First, 2013). Clinical presentations commonly encompass deficits across multiple motor domains, including but not limited to: impaired postural control stability (Sepehri Bonab and Ahmadi, 2025), deficient dynamic balance (Goetschalckx et al., 2024), reduced spatial accuracy, and inefficient motor sequence planning (Pranjić et al., 2023).

Epidemiological data indicate a DCD prevalence of 5%–6% in school-aged children (Steenbergen et al., 2024), with a male-to-female ratio ranging from 2:1 to 7:1 (Blank et al., 2019a). This suggests approximately 1 in 20 children worldwide is affected—a prevalence surpassing many common pediatric chronic conditions. Impaired hand-eye coordination and fine motor skills are widely recognized as core deficits in DCD (Rafique and Northway, 2021; Wuang et al., 2022; Koul et al., 2023). Hand-eye coordination requires precise spatiotemporal integration of visual input and manual output, while fine motor skills depend on highly differentiated control of small hand musculature (Giustino et al., 2023). Studies report that 50% of children with DCD exhibit dysgraphia (Baldi and Caravale, 2018), which may persist into adolescence and adulthood without intervention (Forde and Smyth, 2022). These deficits not only hinder academic performance but may also trigger frustration and social avoidance, perpetuating a “motor difficulty–activity avoidance–skill deterioration” cycle (Kilroy et al., 2022). DCD demonstrates high comorbidity rates, frequently co-occurring with attention-deficit/hyperactivity disorder (ADHD) (Tamplain et al., 2024; Zaguri-Vittenberg et al., 2025), specific learning disorders (e.g., dyslexia, written expression disorder) (Lopez et al., 2018; Visser et al., 2022), speech-language impairments (Reynolds et al., 2015), and emotional-behavioral problems (e.g., anxiety, depression) (Broletti et al., 2024; Gur-Hartman et al., 2024), thereby complicating clinical profiles and intensifying intervention urgency.

With rising global diagnostic rates of pediatric DCD, evidence-based interventions have advanced considerably. Pharmacotherapy, as one approach, has shown progress in efficacy and mechanistic understanding. Methylphenidate—a central nervous system stimulant—enhances fine motor performance and overall coordination in DCD by modulating dopaminergic and noradrenergic systems (Flapper et al., 2006; Flapper and Schoemaker, 2008; Bart et al., 2010). However, pharmacological treatments require prolonged administration, exhibit delayed effects, and carry potential adverse reactions. In contrast, motor interventions, with their physiological adaptability and neuroplasticity induction, present distinct advantages. Common intervention methods are generally divided into two major categories: one is bottom-up process-oriented intervention, which emphasizes the movement process and quality during task execution, aiming to improve physical functions (such as perception, sensory integration, and muscle strength), thereby alleviating activity limitations. Another type is top-down task-oriented intervention, which encourages individuals to use any possible means to complete the ultimate task or goal, emphasizing the improvement of skill acquisition and performance in functional tasks (Yu et al., 2018; Gao et al., 2025). Structured, progressive task-specific training (e.g., rhythmic stepping tasks) rapidly improves coordination and promotes motor automation via enhanced prefrontal-cerebellar network connectivity, offering a safer alternative for DCD management (Al-Yahya et al., 2023). Dynamic gamification in motor interventions also better motivates participation, addressing DCD-related activity avoidance tendencies.

Although numerous studies demonstrate the positive effects of varied motor interventions on gross motor competence in DCD, effect heterogeneity persists. Whether these interventions effectively improve fine motor skills and hand-eye coordination remains debated. However, existing evidence suggests that certain types of exercise interventions may be particularly beneficial. For instance, Amato et al. (2023) demonstrated that basketball training improved fine motor skills such as manual dexterity more effectively than other sports or no sport participation, highlighting the potential of sport-specific motor demands in promoting skill development (Amato et al., 2023). Therefore, this study aims to conduct a meta-analysis of research on exercise interventions for children with DCD, focusing on the dimensions of fine motor skills and hand-eye coordination. It will systematically examine the effects of exercise intensity, intervention duration, and different types of exercise interventions, thus aiming to provide a theoretical basis for developing scientific exercise programs for this population.

## 2 Methods

This systematic review was conducted and reported in accordance with the Preferred Reporting Items for Systematic Reviews and Meta-Analyses (PRISMA) guidelines. Furthermore, the review protocol was registered on the PROSPERO website (Registration number: CRD420251118109).

### 2.1 Literature inclusion criteria

Inclusion criteria were established according to PICOS (Participants, Interventions, Comparisons, Outcomes, Study Design):1) Participants: Children under 12 years old with a confirmed diagnosis of developmental coordination disorder (DCD); 2) Interventions: The intervention measures were any form of physical activity, including but not limited to exercise therapy and sports activities; 3) Comparisons: DCD children not receiving the same physical activity as the experimental group or receiving conventional treatment; 4) Outcomes: Studies providing direct or indirect access to means and standard deviations of hand-eye coordination or fine motor skills in intervention and control groups; 5) Study design: Randomized controlled trials and quasi-experimental studies; 6) Baseline equivalence: No significant differences in baseline data between experimental and control groups before motor intervention; 7) Exercise intensity was classified as low, moderate, or moderate-to-vigorous, with reference to the established ACSM guidelines.

### 2.2 Literature exclusion criteria

Exclusion criteria: 1) Exclusion of patients with multiple comorbid conditions beyond DCD; 2) Literature where original data could not be obtained after exhaustive methods or lacked relevant outcome indicators; 3) Duplicate publications or low-quality literature (when multiple reports existed for the same study, the most recently published was selected); 4) Case studies, review articles, conference papers, and dissertations.

### 2.3 Literature search strategy

Computerized searches were performed across four databases: Web of Science, PubMed, Cochrane Library, and Embase. Subject headings and free-text terms were combined to retrieve literature on exercise interventions improving hand-eye coordination and fine motor skills in DCD children. References of relevant literature were traced to expand search coverage. English search terms included: sport, developmental coordination disorder, children, fine motor skills, hand-eye coordination, etc. Boolean logic operators “OR” and/or “AND” connected search terms. Taking PubMed as an example, the specific search strategy is presented in Table 1. The search period spanned from each database’s inception to 18 July 2025.

**TABLE 1:** PubMed search strategy.

Serial number	Search content
#1	sport OR exercise OR physical activit OR physical exercise OR physical education OR acute exercise OR chronic exercise OR aerobic exercise OR resistance exercise OR exercise intervention OR exergaming OR fitness
#2	DCD OR developmental coordination disorder OR motor skills disorders OR clumsy child syndrome OR motor learning disability
#3	children OR childhood OR school age OR youth preschool OR Preschoolers
#4	fine motor skills OR fine motor OR manual dexterity OR finger movement OR fine control OR hand-eye coordination OR hand function OR visuomotor skill OR visual-motor integration OR movement coordination
#5	#1 and #2 and #3 and #4

### 2.4 Literature screening and data extraction

Two researchers independently conducted literature searches across databases according to the predefined inclusion and exclusion criteria, using Zotero 7.0 to remove duplicate publications. After deduplication, researchers independently screened titles and abstracts of remaining records, excluding obviously irrelevant studies based on the criteria. Full texts of preliminarily selected articles were obtained and thoroughly reviewed. For studies with unavailable data, corresponding authors were contacted via email. Studies remained excluded if data could not be obtained after exhaustive attempts. Two researchers cross-verified inclusion results, with a third researcher resolving disagreements through discussion. Extracted study characteristics included: author names, publication year, sample size, mean age, gender ratio, weekly exercise duration, intervention type, control intervention type, study design, and assessment tools. Pre- and post-intervention means and standard deviations were extracted for mean difference calculation. Following Follmann et al.'s (1992) method, we adopted a conservative approach assuming a correlation coefficient of 0.5 (Follmann et al., 1992). Standardized mean differences (SMDs) with 95% confidence intervals (CIs) were calculated for continuous outcomes. Results were considered statistically significant if the 95% CI excluded zero.

### 2.5 Quality assessment of included studies

Two researchers independently evaluated the methodological quality of included randomized controlled trials and quasi-experimental studies using the Cochrane Risk of Bias Tool (Higgins et al., 2011). Key domains assessed included: random sequence generation, allocation concealment, blinding (of participants and personnel), completeness of outcome data, selective reporting, and other potential biases. Discrepancies in quality ratings were resolved through discussion with a third researcher.

### 2.6 Statistical analysis

RevMan 5.4 and Stata 18.0 were employed for meta-analysis. All outcome measures were continuous variables analyzed using SMDs with 95% CIs. Between-study heterogeneity was quantified using I^2^ statistics: fixed-effect models were applied when I^2^ < 50%, while random-effects models were used for I^2^ ≥ 50% to account for heterogeneity. Effect sizes were interpreted as small (0.2), medium (0.5), or large (0.8). Sensitivity analyses explored heterogeneity sources, with subgroup analyses examining intervention parameters (e.g., intensity, total duration).

Given that 14 studies utilized four different assessment tools (or different versions of the same tool) to measure hand-eye coordination and fine motor skills - with some scales favoring lower scores and others higher scores for better outcomes - we unified effect directions by multiplying means and SDs by −1 for “lower score = better outcome” studies. This ensured all effects consistently reflected “higher scores indicating better outcomes” for interpretability.

Meta-regression analyses in Stata 18.0 examined potential dose-response relationships between effect sizes and continuous intervention parameters (weeks, frequency, session duration). Publication bias was assessed using Egger’s test.

## 3 Results

### 3.1 Literature screening and inclusion results

**FIGURE 1:**
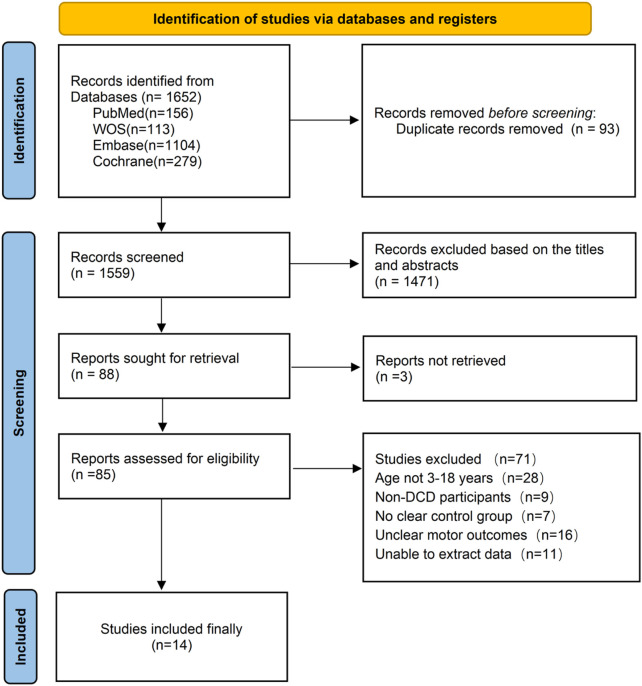
Literature screening and selection Flowchart.

Initial searches across four databases yielded 1,652 records. After applying the predefined inclusion and exclusion criteria, 14 English-language studies were ultimately included. The literature screening process is illustrated in Figure 1.

### 3.2 Basic characteristics of included studies and risk of bias assessment

**FIGURE 2:**
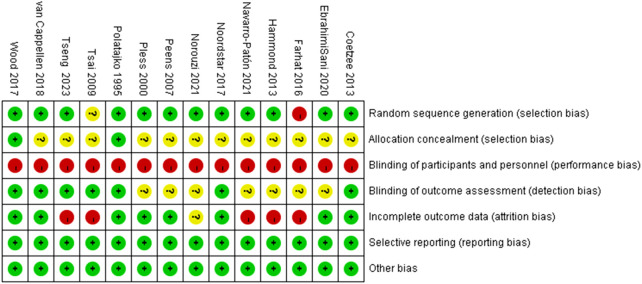
Risk of bias graph for included studies.

**FIGURE 3:**
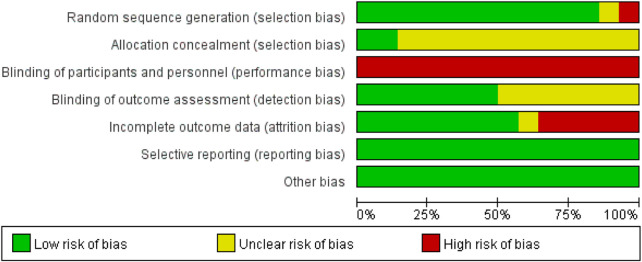
Risk of bias summary graph for included studies.

The 14 included studies involved a total of 528 children with DCD (intervention group: 273; control group: 255). Among these, 12 studies reported post-intervention scores for hand-eye coordination, and 12 studies reported post-intervention scores for fine motor skills. Baseline data showed no statistically significant differences between intervention and control groups across all 14 studies. While the overall quality of the included studies was good, significant heterogeneity was observed in some results. This heterogeneity primarily stems from the difficulty in blinding participants to physical activity interventions, leading to potential performance bias. Additionally, the absence of randomization and allocation concealment in some non-randomized controlled trials may have contributed to selection bias. Detailed characteristics of the included studies are presented in Table 2, with risk of bias assessment results shown in Figures 2, 3.

**TABLE 2:** Characteristics of included studies.

Study	Country	Diagnostic	Study design	(E/C)	Experimental group	Intensity	Intervention does	Outcome indicator
Coetzee and Pienaar (2013a)	South Africa	DSM-IV	RCT	16/16	Visual-motor	LPA	40 m*1t*18w	M-ABC
EbrahimiSani et al. (2020)	Iran	DSM-V	RCT	20/20	Virtual reality	MVPA	30 m*2t*8w	BOT-2
Farhat et al. (2016)	Tunisia	DSM-V	QED	14/13	Comprehensive exercise	MVPA	60 m*3t*8w	M-ABC
Hammond et al. (2014)	United Kingdom	DCD-Q	RCT	10/8	Comprehensive exercise	LPA	10 m*3t*4w	BOT-2
Navarro-Patón et al. (2021)	Spain	M-ABC	QED	12/16	Comprehensive exercise	MVPA	40 m*1t*6w	M-ABC
Noordstar et al. (2017)	Netherlands	DSM-V	QED	20/11	Comprehensive exercise	MPA	30 m*1t*12w	M-ABC
Norouzi Seyed Hosseini et al. (2021)	Iran	M-ABC	RCT	20/20	Visual-motor	MPA	40 m*2t*4w	M-ABC
Peens et al. (2007)	South Africa	DSM-V	RCT	58/58	Comprehensive exercise	MPA	30 m*2t*8w	M-ABC
Pless et al. (2000)	Sweden	DSM-IV	RCT	17/20	Comprehensive exercise	MPA	40 m*1t*10w	M-ABC
Polatajko et al. (1995)	Canada	DSM-III	RCT	26/24	Comprehensive exercise	LPA	20 m*2-3t*5w	VMI、TOMI
Tsai (2009)	Taiwan	M-ABC	RCT	27/16	Table tennis	MPA	50 m*3t*10w	M-ABC
Tseng et al. (2023)	Taiwan	DSM-V	QED	10/10	Table tennis	MPA	40 m*3t*12w	M-ABC
van Cappellen – van Maldegem et al. (2018)	Netherlands	DSM-V	QED	12/13	Throwing exercise	LPA	50 m*2t*3w	M-ABC
Wood et al. (2017)	United Kingdom	M-ABC	RCT	11/10	Visual-motor	LPA	60 m*1t*4w	M-ABC

DSM-III: diagnostic and statistical manual of mental disorders, Third Edition; DSM-IV: diagnostic and statistical manual of mental disorders, Fourth Edition; DSM-V: diagnostic and statistical manual of mental disorders, Fifth Edition; M-ABC: movement assessment battery for children; DCD-Q: developmental coordination disorder questionnaire; RCT: randomized controlled trial; QED: Quasi-Experimental Design; LPA: low physical activity; MPA: moderate physical activity; MVPA: Moderate-to-Vigorous Physical Activity; BOT-2: Bruininks-Oseretsky Test of Motor Proficiency, Second Edition; VMI: Beery-Buktenica Developmental Test of Visual-Motor Integration; TOMI: test of motor impairment; m: minutes; t: sessions per week; w: intervention weeks; (E/C): Experimental group sample size/Control group sample size.

### 3.3 Meta-analysis results

**FIGURE 4:**
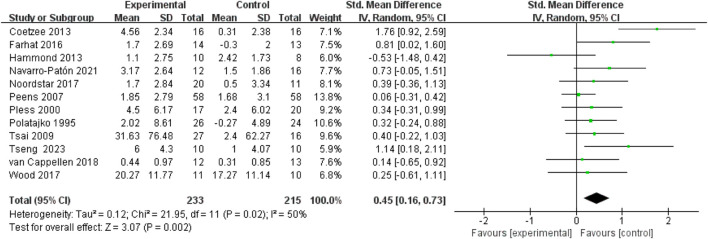
Forest plot of the effect of motor intervention on hand-eye coordination in children with DCD.

**FIGURE 5:**
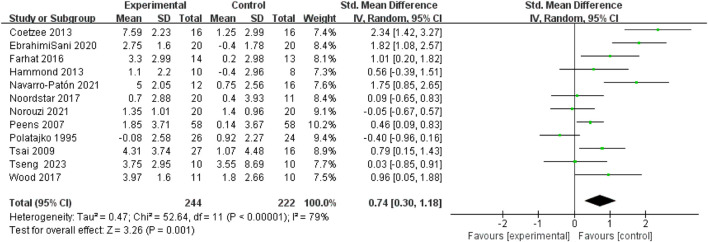
Forest plot of the effect of motor intervention on fine motor skills in children with DCD.

All 14 included studies reported outcome measure scores for hand-eye coordination and fine motor skills post-intervention. The assessment scales incorporated into the analysis comprised the M-ABC, BOT-2, VMI, and TOMI (totaling four types). Consequently, the Standardized Mean Difference (SMD) was utilized for analysis, and data were pooled to calculate effect sizes. The results indicated substantial heterogeneity across studies (I^2^ > 50%) for both outcomes; therefore, random-effects models were employed for all analyses. Separate meta-analyses for hand-eye coordination and fine motor skills revealed that motor interventions yielded a small effect size improvement in hand-eye coordination for children with DCD(SMD = 0.45, 95%CI: 0.16-0.73, P = 0.002) and a moderate effect size improvement in fine motor skills (SMD = 0.74, 95%CI: 0.3-1.18, P = 0.001). Both improvements were statistically significant. See Figures 4, 5.

### 3.4 Subgroup analysis

This study further explored the sources of heterogeneity through subgroup analyses. When exercise intensity was used as the moderator variable, the results showed that low-intensity exercise yielded no significant improvement in hand-eye coordination or fine motor skills (P > 0.05). Moderate-intensity exercise resulted in small effect size improvements in both hand-eye coordination and fine motor skills (P < 0.01). Moderate-to-high intensity exercise yielded a large effect size improvement in fine motor skills (P < 0.001). When total intervention duration was used as the moderator variable, the results showed that a total intervention duration of <500 min yielded a small effect size improvement in fine motor skills (P < 0.05). A total intervention duration of >720 min (typically corresponding to approximately 8–12 weeks, involving 18–36 sessions in the included studies) yielded large effect size improvements in both hand-eye coordination and fine motor skills (P < 0.05). See Table 3.

**TABLE 3:** Subgroup analysis of the effects of exercise Intervention variables on hand-eye coordination and fine motor skills in children with DCD.

Adjusting variable	Subgroup category	Meta-analysis results of hand-eye coordination			Meta-analysis results of fine motor skills		
		SMD (95%CI)	Z	P	SMD (95%CI)	Z	P
Exercise intensity	LPA	0.40 (−0.27–1.06)	1.17	0.24	0.83 (−0.38–2.04)	1.35	0.18
	MPA	0.31 (0.01–0.60)	2.06	0.04	0.33 (0.04–0.62)	2.25	0.02
	MVPA	0.77 (0.22–1.32)	2.73	0.006	1.22 (0.75–1.68)	5.15	<0.001
Total duration of intervention	<500min	0.20 (−0.02–0.42)	1.78	0.07	0.40 (0.12–0.67)	2.24	0.02
	>720min	0.98 (0.39–1.58)	3.24	0.001	1.02 (0.18–1.87)	2.38	0.02

### 3.5 Sensitivity analysis

Sensitivity analysis was performed using the leave-one-out method to examine changes in outcome measures after sequential exclusion of individual studies. The results demonstrated no directional changes upon removal of any single study, indicating minimal influence of individual studies on the pooled effect sizes and confirming the robustness of the meta-analysis results.

### 3.6 Publication bias

**FIGURE 6:**
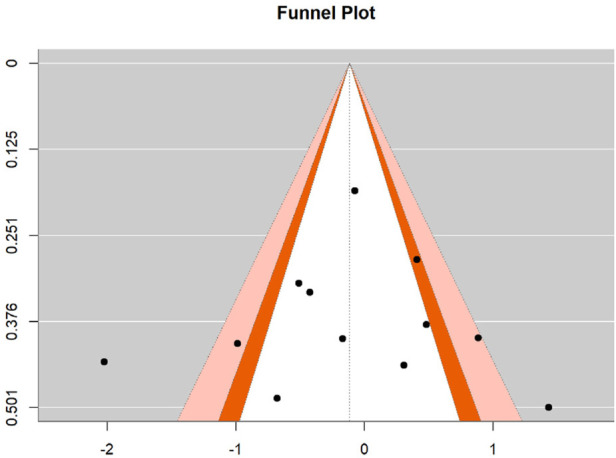
Funnel plot for hand-eye coordination.

**FIGURE 7:**
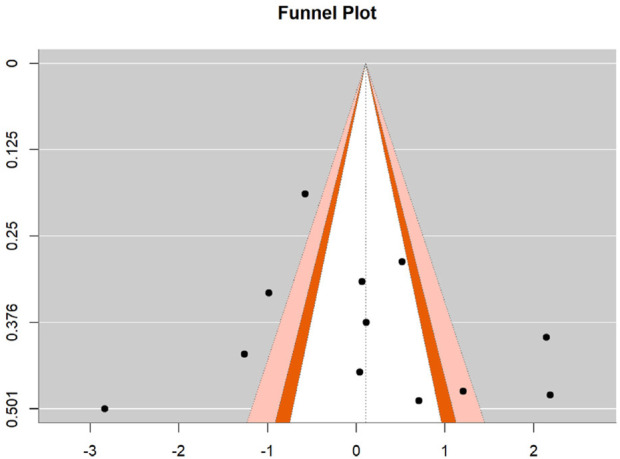
Funnel plot for fine motor skills.

To assess potential publication bias in the effect sizes of motor skill interventions, Egger’s regression test was employed to statistically evaluate funnel plot symmetry for both hand-eye coordination and fine motor skills. For hand-eye coordination, the regression test yielded a z-value of 0.032 (p = 0.974), substantially exceeding the 0.05 significance threshold, indicating satisfactory funnel plot symmetry with no detectable publication bias. The intercept estimate was −0.151 (95% CI: −2.445, 2.142), with the confidence interval encompassing zero. For fine motor skills, the test produced a z-value of 0.330 (p = 0.741), also well above the 0.05 significance level, demonstrating good funnel plot symmetry without significant publication bias. The intercept estimate was −0.478 (95% CI: −4.044, 3.087), similarly containing zero within its confidence interval. These results suggest homogeneous distribution of effect sizes across included studies for both outcomes, with no systematic publication bias related to statistical significance or effect magnitude, thereby strengthening the credibility and external validity of the present meta-analysis. See Figures 6, 7.

### 3.7 Regression analysis

**FIGURE 8:**
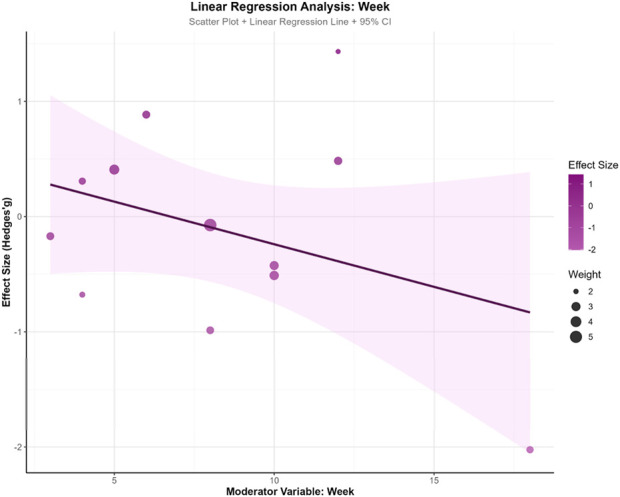
Training weeks.

**FIGURE 9:**
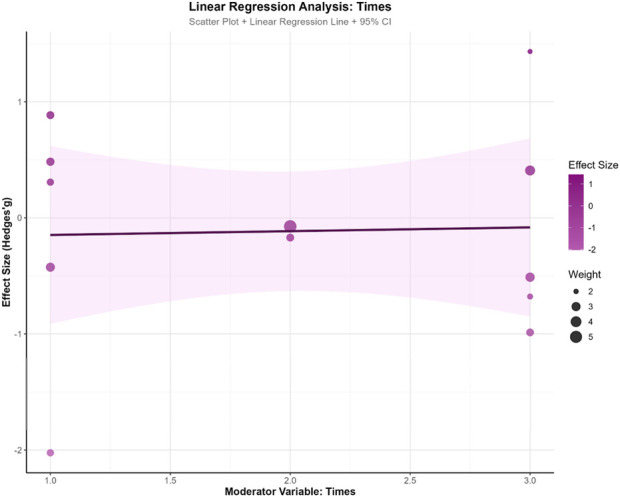
Training frequency.

**FIGURE 10:**
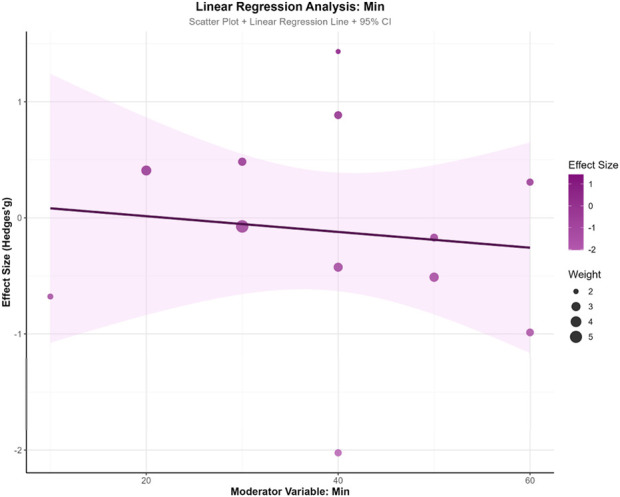
Single-session duration.

To explore the influence of potential moderator variables on exercise intervention effects, this study conducted meta-regression analyses on training weeks (WeekREG), training frequency (TimesREG), and single-session duration (MinREG). The results revealed a marginal trend for training weeks (β = −0.074, SE = 0.059, z = −1.251, p = 0.211, 95% CI: −0.190, 0.042), suggesting a slight decreasing trend in intervention effects with increasing training duration, though this did not reach statistical significance. Training frequency demonstrated minimal influence on effect sizes (β = 0.033, SE = 0.290, z = 0.112, p = 0.911, 95% CI: −0.536, 0.601), indicating no apparent association between weekly sessions and effect magnitude. Single-session duration similarly showed no significant effect (β = −0.007, SE = 0.018, z = −0.371, p = 0.711, 95% CI: −0.043, 0.029).Model fit indices showed τ^2^ = 0 for all three moderator models, indicating partial explanation of between-study heterogeneity by these variables. However, all models demonstrated significant QE tests (p < 0.001), suggesting residual unexplained heterogeneity. In model comparisons, the training weeks model yielded the smallest AIC value (33.586), indicating relatively better model fit. This analysis found no significant moderating effects of training parameters (weeks, frequency, duration) on intervention outcomes, suggesting that effect heterogeneity may originate from other unmeasured factors. See Figures 8–10.

## 4 Discussion

### 4.1 Effect of exercise intervention on hand-eye coordination and fine motor skills in children with DCD

This meta-analysis incorporated 14 studies investigating the effect of exercise intervention on hand-eye coordination and fine motor skills in children with Developmental Coordination Disorder (DCD). The results demonstrate that exercise effectively enhances both hand-eye coordination (SMD = 0.45, P = 0.002) and fine motor skills (SMD = 0.74, P = 0.001) in this population. This finding supports the efficacy of exercise intervention as a first-line intervention strategy for DCD.

Current research posits that the core neural underpinnings of DCD involve dysfunctional coordination within multiple brain networks. These primarily include the cerebellum, responsible for motor coordination, timing control, and error correction; the basal ganglia, involved in action selection and procedural learning; and the sensorimotor cortex, which processes sensory information and executes motor commands (Zwicker et al., 2009; McLeod et al., 2014; 2016). Furthermore, the prefrontal cortex, implicated in higher cognitive functions such as attention, working memory, and action planning, is also associated with DCD deficits (Querne et al., 2008; Fuelscher et al., 2018). Exercise interventions, particularly structured motor training requiring sustained attention and action planning, contribute to strengthening functional connectivity between the prefrontal and motor cortices (Al-Yahya et al., 2023). For instance, tasks demanding precise timing and coordination (e.g., virtual reality tasks) can enhance functional connectivity within the cerebellar-cortical loop, improving movement fluency (Zwicker et al., 2011). Complex coordinative movements can induce neuroplasticity in the sensorimotor cortex, thereby refining the precision of sensory feedback (proprioception, vision) and motor output (Bo and Lee, 2013). Although the present meta-analysis found that exercise interventions yielded small-to-moderate effect size improvements in hand-eye coordination and fine motor skills for children with DCD, significant heterogeneity was observed between studies. To explore the sources of this heterogeneity, further subgroup analyses were conducted.

### 4.2 Subgroup analysis of the effects of exercise intervention variables on hand-eye coordination and fine motor skills in children with DCD

Subgroup analysis revealed that exercise intensity and total intervention duration were key moderator variables determining intervention effectiveness. Subgroup analysis by exercise intensity showed that low-intensity exercise yielded no significant improvement in either hand-eye coordination or fine motor skills (P > 0.05). While moderate-intensity exercise produced small effect size improvements, moderate-to-high intensity exercise yielded the greatest improvement in fine motor skills (SMD = 1.22, P < 0.001). This indicates a differential association between exercise intensity levels and improvements in hand-eye coordination versus fine motor skills. Research suggests that higher-intensity exercise elicits greater activation and connectivity within the cerebellum, basal ganglia, and sensorimotor cortex, promoting neuroplasticity (Hortobágyi et al., 2022). This level of intensity more effectively challenges and remodels the neuromuscular control system, leading to more pronounced improvements in domains requiring high coordination and precision, such as fine motor skills, for children with DCD. Despite the superior efficacy of moderate-to-high intensity, moderate intensity still significantly improved fine motor skills (SMD = 0.33, P = 0.02), potentially offering a viable option for children with lower tolerance, warranting further investigation.

Subgroup analysis by total intervention duration showed that a cumulative duration of <500 min resulted in a small effect size improvement for fine motor skills, whereas durations exceeding 720 min (typically achieved over 8–12 weeks with 18–36 sessions) yielded large effect size improvements in both hand-eye coordination (SMD = 0.98, P = 0.001) and fine motor skills (SMD = 1.02, P = 0.02). This may be attributed to the progressive process of skill automatization, which requires sufficient time and practice repetitions. Short-term interventions may only induce initial adaptations or strategic changes, insufficient for deep neural circuit reorganization and skill consolidation. This underscores the need to ensure total intervention duration reaches a minimum effective threshold (e.g., >720 min) in clinical training protocols to achieve substantial skill improvement. Furthermore, the varying degrees of impairment in hand-eye coordination and fine motor skills among the included children with DCD, coupled with a wide age range, may have introduced bias into the results, necessitating future research for validation.

It is noteworthy that the included studies encompassed diverse exercise intervention types (e.g., comprehensive exercise, visual-motor training, table tennis, virtual reality, throwing). Although the subgroup analysis primarily focused on parameters like intensity and duration, the type of exercise itself may also be a significant factor influencing outcomes. According to the “principle of specificity,” training effects transfer maximally to contexts similar to the training task itself. For instance, task-oriented training like virtual reality or table tennis might offer advantages over general comprehensive exercise through specific neuroplastic mechanisms. Future research should more meticulously analyze the relative benefits of different exercise modalities (e.g., open vs closed motor skills, ball sports vs non-apparatus activities) on specific skills (hand-eye coordination vs fine motor skills) in children with DCD. This will provide more direct evidence for designing individualized, precise intervention plans.

### 4.3 Influence of different types of exercise interventions on hand-eye coordination and fine motor skills in children with DCD

Different types of motor intervention, through their unique activity patterns, can effectively stimulate the neuromuscular control system of children with DCD. Although subgroup analysis mainly focused on parameters such as intensity and duration, the type of exercise itself might also be an important factor influencing the outcome.

#### 4.3.1 Process-oriented intervention

Process-oriented intervention is a method that applies activities to address underlying behavioral problems (Blank et al., 2019b), aiming to help children correct sensory deficits by providing proprioceptive, tactile, kinesthetic, and vestibular stimulation. For children with DCD, clumsiness in hand-eye coordination and fine motor skills such as writing and buttoning is essentially the external manifestation of internal process dysfunctions like perceptual information processing and motor planning programming. Therefore, directly strengthening these weak foundational processes through sensory integration training and perceptual-motor therapy can provide more solid support for the execution of hand-eye coordination and fine motor skills.

In the study by Peens et al. (2007), the intervention group received 8 weeks of sensory integration training, twice weekly for 30 min per session, covering ball skills, balance ability, fine motor skills, and eye movement control. The results showed that sensory integration training not only improved the children’s overall motor ability but also showed positive trends in fine motor skills. Tsai (2009) conducted table tennis training for 10 weeks, three times per week, 50 min per session. The results showed significant improvements in both hand-eye coordination and fine motor skills in children with DCD post-intervention. Coetzee and Pienaar (2013b) implemented visual therapy for 32 children with DCD aged 7–8 years over 18 weeks, once weekly for 40 min per session. The training included tasks such as monocular and binocular tracking, fixation, vergence, combined with balance and hand-eye coordination activities. Results indicated significant improvements in hand agility and visual tracking post-intervention, with effects remaining significant 2 years later. Similarly, Wood et al. (2017) used quiet eye training for 4 weeks, once weekly for 60 min per session. The results showed that the intervention group significantly outperformed the traditional technical training group in eye control, movement coordination, and catching success rate.

However, not all studies reported consistently positive results. Norouzi Seyed Hosseini et al. (2021) adopted quiet eye training twice a week for 40 min each time for 4 weeks. The results showed that there was no significant improvement in fine motor skills. van Cappellen–van Maldegem et al. (2018) focused on throwing skills learning. Through an intervention twice a week for 50 min each time for 3 weeks, the results showed that there was no significant improvement in hand-eye coordination in children. Polatajko et al. (1995) evaluated fine motor skills through a sub-item of the “Sports Injury Test” by conducting process guidance therapy 2-3 times a week for 45 min each time for 9 weeks. The results showed that there was no improvement in fine motor skills either after the intervention or at the 6-week follow-up. Tseng et al. (2023) used table tennis as an intervention method to train children with DCD for 12 weeks, three times a week, for 40 min each time. The results showed that the experimental group had significant improvements in hand-eye coordination and motor function, but the effect was not significant in fine motor skills. Noordstar et al. (2017) conducted a 12-week training program, once a week for 30 min each time, covering ball games, balance and fine motor tasks. The results showed that neither the intervention group nor the conventional intervention group witnessed significant improvement in fine motor skills in the children.

In summary, the effects of process-oriented intervention on improving hand-eye coordination and fine motor skills in children with DCD are contradictory. Some studies show significant and lasting improvements in overall motor ability, hand dexterity, and visual tracking; however, other studies found that improvements in core issues like hand-eye coordination and fine motor skills failed to reach significance. This heterogeneity in effects suggests that the actual benefits of process-oriented intervention may vary depending on the intervention content, assessment methods, or individual differences among children, and a consensus on its efficacy has not been reached. Compared to process-oriented intervention, task-oriented intervention is generally considered a more effective and often prioritized mainstream method.

#### 4.3.2 Task-oriented intervention

Task-oriented intervention is a treatment method aimed at resolving the behavioral problem itself (Blank et al., 2019b). This method is goal-centered, and its core lies in optimizing motor control through repetitive practice of functional tasks and situational adjustments, emphasizing the analysis of tasks and adaptive strategies in real environments to alleviate related symptoms in children with DCD and improve their quality of life (Kim et al., 2024).

Navarro-Patón et al. (2021) conducted a specific task intervention once a week for 40 min each time for 6 weeks on children aged four to 6 with DCD. The intervention included manual flexibility tasks in the form of games. The results showed that the scores of hand-eye coordination and fine motor skills in the intervention group were significantly improved. Pless et al. (Pless et al., 2000) conducted a 10-week group motor skills intervention on children aged five to six with DCD. The results showed that the fine motor skills of the children with DCD improved after the intervention. Farhat et al. (Farhat et al., 2016) conducted neuro-motor task training for 14 children with DCD for 8 weeks, three times a week, each session lasting 60 min. The training included basic motor skills such as jumping, throwing, and balancing. The results show that the training not only significantly enhanced children’s gross motor coordination but also improved their fine motor skills without specialized training.

Hammond et al. (2014) used Wii Fit exergaming for practice three times per week, 10 min per session, over 4 weeks. The results showed clear progress in the precision and integration of fine motor skills in some children. However, due to the short intervention period, no improvement was observed in hand-eye coordination, and its long-term impact on hand-eye coordination still requires further verification. Similarly, EbrahimiSani et al. (2020) used virtual reality exercise intervention with Xbox 360 Kinect, conducting training for 40 girls with DCD aged 7–10 years over 8 weeks, twice weekly for 30 min per session. The results showed significant improvement in the intervention group’s performance on three fine motor tasks (hand rotation, inserting a sword, and rotary pursuit), and the training effects were maintained 2 months later. This suggests that VR-based motor imagery training can not only enhance motor imagery ability in children with DCD but also provide an effective pathway for improving their hand-eye coordination and fine motor control.

In summary, task-oriented intervention demonstrates significant effects in improving hand-eye coordination and fine motor skills in children with DCD. Most studies confirm that through repetitive practice of functional tasks, children make progress not only in the directly trained skills, but some studies also observe the generalization of intervention effects to untrained tasks, showing good sustainability. Even when using new technologies like virtual reality, positive potential is shown.

### 4.4 Study limitations

This study has several limitations: 1) The differential prevalence of DCD between sexes may influence intervention effects, but analysis of sex as a moderator was precluded by limitations in the primary data; 2) The relatively small sample sizes of included studies and the incomplete consistency in assessment scales used for hand-eye coordination and fine motor skills may limit the statistical power of the meta-analysis; 3) Only English-language studies were included during screening, potentially introducing selection bias by excluding research in other languages.

### 4.5 Conclusion

This meta-analysis evaluated the effects of exercise intervention on hand-eye coordination and fine motor skills in children with DCD. The results indicate that exercise intervention effectively improves both skills in this population. Among the included studies, interventions characterized by moderate-to-high intensity and a total duration exceeding 720 min (typically achieved over 8–12 weeks with 18–36 sessions) demonstrated more significant effects. However, due to limitations inherent in the current body of research, future studies incorporating more literature are needed to analyze and account for various influencing factors. Further investigation is required to validate the efficacy of exercise interventions for children with DCD and to determine the optimal intervention protocols.

## Data Availability

The original contributions presented in the study are included in the article/supplementary material, further inquiries can be directed to the corresponding author.
